# Cross-Scale Coupling Model of CPFEM and Thermo-Elasto-Plastic FEM for Residual Stress Prediction in TA15 Welds

**DOI:** 10.3390/ma19040754

**Published:** 2026-02-14

**Authors:** Xuezhi Zhang, Yilai Chen, Anguo Huang, Shengyong Pang, Lvjie Liang

**Affiliations:** 1 School of Mechanical and Electrical Engineering, Wuhan Institute of Technology, Wuhan 430205, China; 2 Hubei Research Center of Intelligent Welding Equipment and Software Engineering Technology, Wuhan 430205, China; 3 State Key Laboratory of Materials Processing and Die & Mould Technology, School of Materials Science and Engineering, Huazhong University of Science and Technology, Wuhan 430074, China

**Keywords:** RVE, crystal plasticity finite element model, numerical simulation, TA15 alloy, electron-beam welding

## Abstract

Existing macroscopic finite element models for electron beam welding (EBW) typically assume isotropic material behavior, often failing to accurately predict residual stresses induced by strong crystallographic textures. To address this limitation, this study established a sequential dual-scale coupled numerical model bridging micro-texture to macro-mechanics by combining the crystal plasticity finite element method (CPFEM) with thermal-elastic-plastic theory. Representative volume elements (RVEs) incorporating *α* and *β* dual-phase characteristics were constructed based on electron backscatter diffraction (EBSD) data from the TA15 weld cross-section. Through simulated tensile and shear calculations on the RVEs, homogenized orthotropic stiffness matrices and Hill yield constitutive parameters were derived and mapped onto the macroscopic model. Simulation results indicate that the proposed model maintains the prediction error for molten pool morphology within 16.3%, while effectively correcting the stress overestimation inherent in isotropic models. Specifically, it adjusts the peak longitudinal residual stress at the weld center from 800 MPa to approximately 350 MPa, significantly reducing the anomalous “M-shaped” stress distribution. By successfully capturing shear stress components, this work provides a high-fidelity computational approach for predicting complex stress states in welded joints, offering critical insights for structural integrity assessment.

## 1. Introduction

TA15 titanium alloy is characterized by superior high-temperature performance, high specific strength, excellent thermal processing stability, weldability, and corrosion resistance. Consequently, it is extensively utilized in the aerospace sector for manufacturing critical structural components, engine disks, and blades [1,2,3]. The service performance of titanium alloy welds is contingent upon the distribution of local stress and strain at the grain scale [4]. Specifically, the non-uniform distribution of local stresses and strains, typically induced by the anisotropic slip motion of grains, may lead to the initiation of welding cracks. Due to the transient and localized nature of the electron beam welding process, coupled with complex physical transformations, real-time monitoring of the internal stress field remains challenging in industrial settings. Consequently, numerical simulation emerged as a critical tool for predicting welding stresses from the late 20th century.

The CPFEM has been widely applied to the study of plastic deformation behaviors across different scales [5,6]. Shaolong Li et al. [7] conducted an in-depth analysis of the softening mechanisms and failure behaviors of the high-temperature titanium alloy TA15 at near-service temperatures, specifically investigating the deformation characteristics of the α- and β-phases during both elastic and plastic stages. Balasubramanian et al. [8] utilized CPFEM to study the tensile, compressive, and thin-walled tubular torsional behaviors of commercially pure titanium at 750 °C. They measured the macroscopic stress–strain response and crystallographic texture evolution, subsequently developing a crystal plasticity-based constitutive model. Hongwei Li et al. [9] developed a crystal plasticity model suitable for the dynamic recrystallization (DRX) of dual-phase titanium alloys, which can capture the DRX and thermomechanical behaviors of titanium alloys during processing. Despite the significant progress that CPFEM has made in multiscale modeling successfully establishing the link between microstructure and mechanical properties, the direct application of full-field coupled CPFEM to the transient simulation of large-scale electron beam welded structures remains extremely challenging due to its prohibitive computational costs.

On the other hand, finite element analysis methods grounded in thermo-elasto-plastic theory have been extensively applied in welding simulations. Chuan Liu et al. [10] utilized a hybrid 3D and 2D finite element simulation approach to investigate the residual stress in 50 mm thick TA15 titanium plates subjected to EBW. Peihao Geng et al. [11] developed a sequentially coupled computational modeling method based on 5052 aluminum alloy and DP590 steel to predict the temperature field, residual stress distribution, and warpage deformation. J. Ding et al. [12] evaluated the computational time of steady-state versus transient thermal-displacement sequentially coupled models and compared the simulated residual stress values with measurements obtained via a neutron diffraction strain scanner.

However, existing macroscopic thermo-elasto-plastic finite element simulations still encounter accuracy bottlenecks when addressing weld seams characterized by complex microstructures. Although phenomenological anisotropic models are relatively mature, accurately acquiring local anisotropic parameters induced by crystallographic texture evolution within the weld zone remains extremely difficult. Consequently, to prioritize computational efficiency, the majority of engineering thermo-elasto-plastic analyses simplify the material representation to an isotropic model, potentially overlooking unique stress coupling effects driven by the microstructure.

To address these issues, this paper proposes a stress simulation algorithm for electron beam welding based on the CPFEM. Through a sequential coupling strategy, the proposed algorithm utilizes CPFEM to solve homogenized material constitutive equations that reflect micro-texture features and maps them into the macroscopic model, thereby achieving a numerical simulation of welding that reconciles micro-mechanical accuracy with macro-computational efficiency.

## 2. The Model

### 2.1. Overall

**Figure 1:**
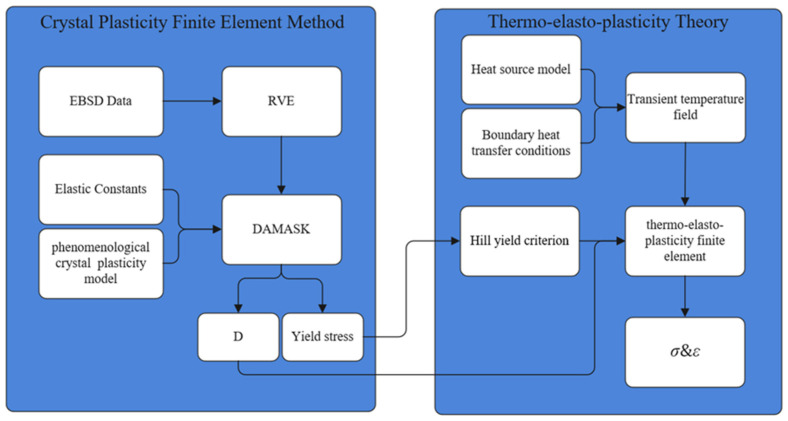
Stress simulation algorithm for EBW based on the CPFEM.

The stress simulation algorithm for EBW of TA15 material, based on the CPFEM, can be divided into two computational modules, as shown in Figure 1.

The CPFEM module (left panel of Figure 1) establishes the micro-scale constitutive relationship. It begins with the construction of a representative volume element (RVE) derived from experimental EBSD data. This RVE incorporates data regarding crystal orientation, as well as the spatial distribution and volume fractions of the phases. Within the DAMASK finite element software environment, the RVE serves as the basis for assigning corresponding single-crystal elastic stiffness constants and phenomenological crystal plasticity constitutive parameters to the different phases. The solver utilizes a fast Fourier transform (FFT)-based full-field crystal plasticity spectral method to perform uniaxial tensile and pure shear calculations on the RVE. Subsequently, the results are processed using the volume averaging method to derive the homogenized orthotropic stiffness matrix, D, and the yield stress from the stress–strain curves. The CPFEM calculation encompasses the complete elasto-plastic response based on slip systems; within the multi-scale framework; it is employed to capture the anisotropic elastic and plastic characteristics induced by the specific crystallographic texture of the weld zone, thereby providing the necessary elastic and plastic parameters for the macroscopic model.

The macroscopic module (right panel of Figure 1) employs the thermo-elasto-plastic finite element framework. This module utilizes a sequential thermal–mechanical coupling strategy, where both elasticity and plasticity are governed by anisotropic constitutive relations derived from the CPFEM results. In this module, both elasticity and plasticity are governed by anisotropic constitutive relations. The core mechanism involves utilizing the homogenized orthotropic stiffness matrix, D, and the yield stress data derived from the CPFEM module to transfer the microscopic texture characteristics of the weld zone into macroscopic thermo-elasto-plastic constitutive equations. Within this framework, the response relationship between stress increments and elastic strain increments adheres to the anisotropic Hooke’s law, while the plastic yield behavior is governed by the Hill criterion. This strategy ensures that the heterogeneity of the mechanical response induced by crystallographic texture is captured across both elastic stiffness and plastic yield dimensions, thereby overcoming the limitations in prediction accuracy associated with isotropic models.

### 2.2. Calculation of Macroscopic Anisotropic Stiffness Matrix via CPFEM

The crystal plasticity simulation is performed in the DAMASK software (V3.0.2) [13], with its theoretical framework based on the large-deformation and rate-dependent crystal plasticity theory proposed in the literature [14,15].

In this theory, plastic deformation within the crystal is caused entirely by shear flow on the slip systems. The shear deformation on the slip planes is induced by the generation and motion of dislocations. Its kinematics are based on the multiplicative decomposition of the total deformation gradient *F*:

(1) $$F=F_{e}F_{p}$$

$F_{e}$ is the elastic deformation part, which includes elastic lattice stretching and rigid body rotation, and $F_{p}$ is the plastic deformation and rotation caused by shear in multiple slip systems.

According to the generalized Hook’s law [13], in the elastic part, stress and strain are approximated as follows:

(2) $$S=C:\left(F_{e}^{T}F_{e}-I\right)/2$$

where S denotes the second Piola–Kirchhoff stress tensor, and C represents the fourth-order elastic stiffness tensor. For the α-phase in the TA15 alloy, governed by the intrinsic symmetry of the hexagonal crystal system, the single crystal exhibits transverse isotropy; consequently, its stiffness tensor C comprises five independent elastic constants: $C_{11}{,C}_{12}{,C}_{13}{,C}_{33},$ and $C_{44}$. Regarding the β-phase, consistent with the intrinsic symmetry of the cubic crystal system, the stiffness tensor C is defined by three independent elastic constants: $C_{11}{,C}_{12}$, and $C_{44}$. Based on the aforementioned crystallographic symmetries, the crystal plasticity constitutive model was formulated.

The plastic deformation gradient $F_{p}$ is obtained through the integration of the shear strain rates $\dot{\gamma }$ on the respective slip systems i. For a crystal possessing $N_{sys}$ slip systems, it is calculated using the following formula [16,17]:

(3) $${\dot{F}}_{p}F_{p}^{-1}=\Sigma_{i}^{N_{sys}}{\dot{\gamma }}^{i}(s_{s}^{i}⊗n_{s}^{i})$$

**Figure 2:**
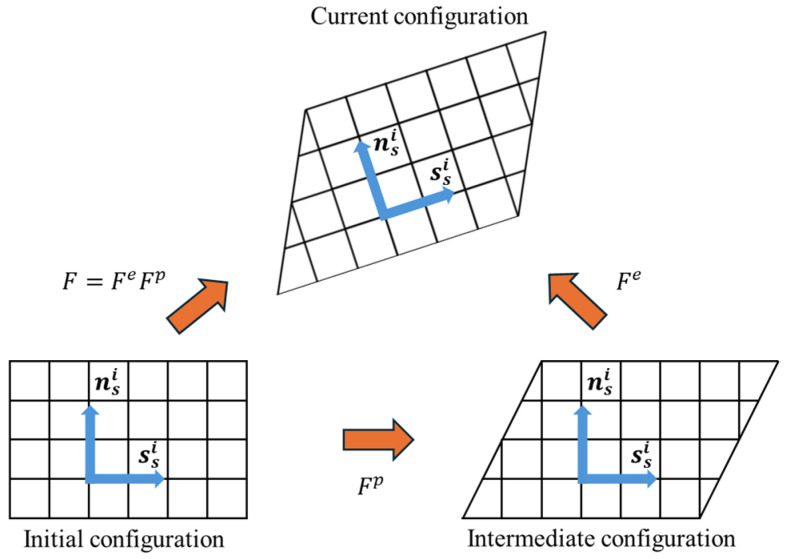
Multiplicative decomposition of the deformation gradient tensor.

As shown in Figure 2, $s_{s}^{i}$ and $n_{s}^{i}$ respectively represent the unit vector along the slip direction and the normal slip plane. For each slip system, the slip rate is governed by a phenomenological flow relation [18], as follows:

(4) $${\dot{\gamma }}^{i}={\dot{\gamma }}_{0}^{i}{\left|\frac{\tau^{i}}{\xi^{i}}\right|}^{n}sgn(\tau^{i})$$

${\dot{\gamma }}_{0}^{i}$ is the reference shear strain rate; n is the power law exponent; $\xi^{i}$ is the slip resistance. In the DAMASK tensile simulation, the evolution of the slip resistance $\xi^{i}$ is incorporated. This resistance evolves from an initial value $\xi_{0}^{j}$ to a saturation value $\xi_{\infty }^{j}$ according to the following relationship [19]:

(5) $${\overset{.}{\xi }}^{i}=h_{0}(1+h_{\mathrm{int}}^{i})\times \Sigma_{i=1}^{N_{sys}}\left|{\overset{.}{\gamma }}^{j}\right|{\left|1-\frac{\xi^{j}}{\xi_{\infty }^{j}}\right|}^{a-1}\left(1-\frac{\xi^{j}}{\xi_{\infty }^{j}}\right)h^{ij}$$

$h_{0}$ is the overall hardening parameter; the dimensionless parameter $h_{\mathrm{int}}^{i}$ is a correction value for $h_{0}$ in a specific slip system. The interaction between different slip systems is characterized by the dimensionless factor $h^{ij}$.

Uniaxial tensile loads along the three principal axes and pure shear loads along the three shear planes were respectively applied to the RVE model to obtain stress–strain response curves under six specific loading conditions. The elastic constants—specifically the Young’s moduli $E_{ij}$ and shear moduli $G_{ij}$ along the three directions—were determined via linear regression analysis of the elastic regime of the stress–strain curves. Regarding the calculation of Poisson’s ratios $v_{ij}$, considering the variations in crystallographic orientation and stiffness among different grains, the local displacement fields within the RVE and at its boundaries exhibit non-uniform characteristics. Consequently, the macroscopic strain was calculated as the volume average of the strains at all integration points within the RVE. Based on this homogenized macroscopic strain data, the Poisson’s ratios for each direction were derived by performing linear regression between the transverse and longitudinal strains within the elastic stage.

It is important to note that while the constituent α-phase single crystals of TA15 are inherently transversely isotropic, the material exhibits orthotropic behavior at the macroscopic level due to the pronounced columnar grain texture within the weld zone. In this study, by performing full-field homogenization on an RVE incorporating authentic texture data, the macroscopic orthotropic elastic constitutive equation was derived. The resulting stiffness matrix is presented in Equation (29).

Substitute the $E_{ij}$, $G_{ij},$ and $v_{ij}$ into the formula:

(6) {ε}=[S]{σ}.

[S] is the flexibility matrix. The stiffness matrix is the inverse of the flexibility matrix:

(7) $$[D]=[S{]}^{-1}$$

Through the transformation via Equation (7), the homogenized macroscopic anisotropic stiffness matrix based on the RVE and CPFEM is derived.

### 2.3. Finite Element Algorithm Based on Thermo-Elasto-Plastic Theory

**Figure 3:**
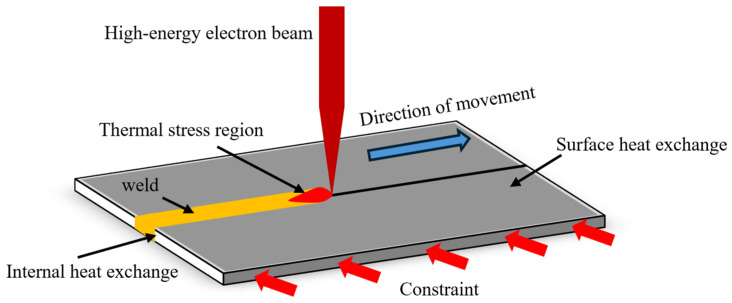
Schematic diagram of EBW.

As shown in Figure 3, the thermo-elastic-plastic theory for the EBW process is based on a macroscopic mathematical model describing temperature, stress, and strain. As the heat source moves, heat propagates outward from the molten pool center via thermal conduction. Simultaneously with heat accumulation, a portion of the heat is dissipated through convective and radiative heat exchange between the workpiece surface and the air; phenomena of the latent heat also accompany melting and phase transformations. Owing to the metallic thermal expansion and contraction effect, local stresses are induced within the workpiece during the subsequent cooling phase of the welding process. In certain regions, the material attains a plastic state and undergoes plastic deformation, culminating in permanent deformation and the formation of residual stress upon the completion of welding and cooling.

The solution for the transient temperature field comprises two primary components: the heat conduction model and the temperature boundary conditions. During the EBW process, the internal heat conduction model satisfies the following equation [20]:

(8) $$c\rho \frac{\partial T}{\partial t}=\frac{\partial }{\partial x}\left(k_{x}\frac{\partial T}{\partial x}\right)+\frac{\partial }{\partial y}\left(k_{y}\frac{\partial T}{\partial y}\right)+\frac{\partial }{\partial z}\left(k_{z}\frac{\partial T}{\partial z}\right)+Q$$

$k_{x}{,\;k}_{y}$, and $k_{z}$ represent the thermal conductivity of the material in the x, y, and z directions, respectively; *c* is the specific heat capacity of the material; *ρ* is the density of the material; *Q* is the input heat source.

**Figure 4:**
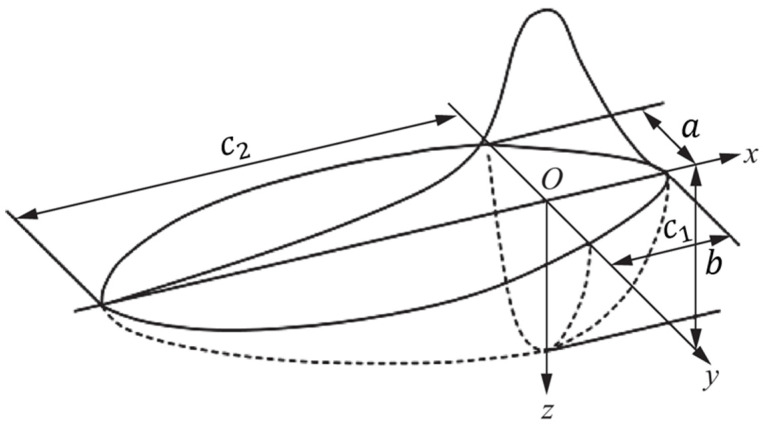
Double-ellipsoid heat source configuration.

During the EBW process, the temperature gradient is steep in front of the heat source center, while the gradient change at the rear tends to be gentler. Based on these heat source characteristics, the heat source model for the weld joint region adopts the double-ellipsoidal model proposed by Goldak [21]. As illustrated in Figure 4, this model’s heat flux exhibits a Gaussian distribution along the longitudinal axis. The model assumes that the front half of the heat source model is one-quarter of an ellipsoid, and the rear half is another one-quarter of an ellipsoid. The power density distribution for the front ellipsoid is as follows:

(9) $$q_{f}(x,y,z)=\frac{6\sqrt{3}f_{f}Q}{abc_{1}\pi \sqrt{\pi }}e^{-3x^{2}/a^{2}}e^{-3y^{2}/b^{2}}e^{-3z^{2}/c_{1}^{2}}$$

The power density distribution for the posterior half-ellipsoid is given by the following:

(10) $$q_{r}(x,y,z)=\frac{6\sqrt{3}f_{r}Q}{abc_{2}\pi \sqrt{\pi }}e^{-3x^{2}/a^{2}}e^{-3y^{2}/b^{2}}e^{-3z^{2}/c_{2}^{2}}$$

$a,\;b,\;c_{1},\;c_{2}$ are the shape parameters of the double-ellipsoidal heat source model; *Q* is the heat input; $f_{f}$ is the energy distribution fraction for the front half-ellipsoid; $f_{r}$ is the energy distribution fraction for the rear half-ellipsoid.

The heat transfer boundary conditions for the plate satisfy the equation [22]:

(11) $$k_{x}\frac{\partial T}{\partial x}n_{x}+k_{y}\frac{\partial T}{\partial y}n_{y}+k_{z}\frac{\partial T}{\partial z}n_{z}=h(T_{env}-T)$$

$n_{x}$, $n_{y}$, $n_{z}$ are the direction cosines of the outward normal vector to the heat transfer boundary; h is the heat transfer coefficient. The effects of both convective and radiative heat transfer are represented by adjusting the numerical value of this coefficient.

Regarding the yield criterion, the Hill yield criterion is employed to determine the yield condition:

(12) $$F{\left(\sigma_{22\;}-\sigma_{33\;}\right)}^{2}+G{\left(\sigma_{33\;}-\sigma_{11\;}\right)}^{2}+H{\left(\sigma_{11\;}-\sigma_{22\;}\right)}^{2}+2L{\sigma_{23}}^{2}+2M{\sigma_{31}}^{2}+2N{\sigma_{12}}^{2}=1$$

where $\sigma_{ij}$ represents the stress components, and the calculation formulas for *F*, *G*, *H*, *L*, *M*, and *N* are as follows:

(13) $$F=\frac{1}{2}\left[\frac{1}{{\left(\sigma_{2}^{y}\right)}^{2}}+\frac{1}{{\left(\sigma_{3}^{y}\right)}^{2}}-\frac{1}{{\left(\sigma_{1}^{y}\right)}^{2}}\right]$$

(14) $$G=\frac{1}{2}\left[\frac{1}{{\left(\sigma_{3}^{y}\right)}^{2}}+\frac{1}{{\left(\sigma_{1}^{y}\right)}^{2}}-\frac{1}{{\left(\sigma_{2}^{y}\right)}^{2}}\right]$$

(15) $$H=\frac{1}{2}\left[\frac{1}{{\left(\sigma_{1}^{y}\right)}^{2}}+\frac{1}{{\left(\sigma_{2}^{y}\right)}^{2}}-\frac{1}{{\left(\sigma_{3}^{y}\right)}^{2}}\right]$$

(16) $$L=\frac{1}{2{\left(\tau_{23}^{y}\right)}^{2}}$$

(17) $$M=\frac{1}{2{\left(\tau_{31}^{y}\right)}^{2}}$$

(18) $$N=\frac{1}{2{\left(\tau_{12}^{y}\right)}^{2}}$$

where $\sigma_{1}^{y}$, $\sigma_{2}^{y}$, and $\sigma_{3}^{y}$ denote the normal yield stresses, and $\tau_{12}^{y}$, $\tau_{23}^{y}$, and $\tau_{31}^{y}$ denote the shear yield stresses.

For the stress field solution, The stress–strain relationship is as follows [23]:

(19) $$\{d\sigma \}=\left[D^{P}\right]\{d\varepsilon \}-\left[D^{P}\right]\{\alpha \}dT+\left[D^{P}\right]\frac{1}{E}\frac{dE}{dT}{\left[D^{e}\right]}^{-1}\{d\sigma \}dT$$

where $\left[D^{P}\right]$ is the plasticity matrix, $\left[D^{e}\right]$ is the elasticity matrix, {α} is the material’s coefficient of linear thermal expansion matrix, and *E* represents the Young’s modulus.

When the material is in the elastic stage

(20) $$\left[D^{P}\right]=\left[D^{e}\right]=\left[D\right]$$

When the material is in the plastic stage

(21) $$\left[D^{e}\right]=\left[D\right]$$

(22) $$\left[D^{P}\right]=\left[D\right]-\frac{\left[D\right]\left\{\frac{\partial F}{\partial \sigma }\right\}{\left\{\frac{\partial F}{\partial \sigma }\right\}}^{T}\left[D\right]}{\left[S\right]}$$

(23) $$[S]={\left[D\right]}^{-1}\frac{\partial \left[D\right]}{\partial T}dT\left[D\right]$$

Regarding the temperature dependence of anisotropic parameters, this study assumes that the degree of anisotropy induced by grain texture remains relatively stable during the thermal cycle. Consequently, while the overall stiffness and strength of the material degrade with increasing temperature, the ratios of yield strengths in different directions are assumed to remain constant.

Based on the temperature-dependent curves, the anisotropic parameters at room temperature are dynamically mapped. In the elastic stage, the stiffness matrix *D*(*T*) at an arbitrary temperature *T* is modified from the room-temperature stiffness matrix using an elastic modulus degradation factor:

(24) $$[D(T)]=[D_{25}]\times \frac{E(T)}{E_{25}}$$

where ${[D}_{25}]$ denotes the stiffness matrix at room temperature, $E_{25}$ represents the elastic modulus at room temperature, and *E*(*T*) is the parameter describing the temperature-dependent variation of the elastic modulus.

For the plastic stage, the evolution of the anisotropic coefficients *F*, *G*, *H*, *L*, *M*, and *N* in Hill’s yield criterion is scaled by the yield strength $\sigma_{y}(T)$. Defining the dimensionless anisotropic yield ratios $R_{ii}$ and $R_{ij}$ and assuming a reference stress $\sigma_{ref}$, the normal component $R_{ii}$ is expressed as follows:

(25) $$R_{ii}=\frac{\sigma_{i}^{y}}{\sigma_{ref}}$$

The shear component $R_{ij}$ is expressed as follows:

(26) $$R_{ij}=\frac{\sqrt{3}\cdot \sigma_{ij}^{y}}{\sigma_{ref}}$$

Taking the normal parameter *F* as an example, and based on Equation (13), the temperature-dependent yield stress is expressed as $\sigma_{ii}^{y}\left(T\right)=R_{ii}\cdot \sigma_{y}\left(T\right)$, yielding the temperature mapping equation for *F*:

(27) $$F(T)=\frac{1}{2}\left(\frac{1}{{R_{22}}^{2}}+\frac{1}{{R_{33}}^{2}}-\frac{1}{{R_{11}}^{2}}\right)\times \frac{1}{{\sigma_{y}(T)}^{2}}$$

Similarly, the temperature mapping equation. for the shear parameter *L* is given by the following:

(28) $$L(T)=\frac{3}{2R_{23}^{2}{\sigma_{y}(T)}^{2}}$$

## 3. Numerical Simulation of EBW of TA15 Alloy

### 3.1. TA15 Material Properties

In this study, the TA15 titanium alloy is selected as the research object. TA15 is a near-α titanium alloy characterized by excellent comprehensive mechanical properties, thermal stability, and weldability, with its chemical composition detailed in Table 1. At the microscopic scale, TA15 is an α + β dual-phase titanium alloy, comprising an α-phase volume fraction of approximately 97.86% and a β-phase volume fraction of approximately 2.14% [7]. Although the matrix α-phase possesses a hexagonal close-packed (HCP) crystal structure, the material exhibits significant macroscopic orthotropic anisotropy due to the preferred grain orientation induced by the welding thermal cycles.

### 3.2. Crystal Plasticity Finite Element Parameter Construction

**Figure 5:**
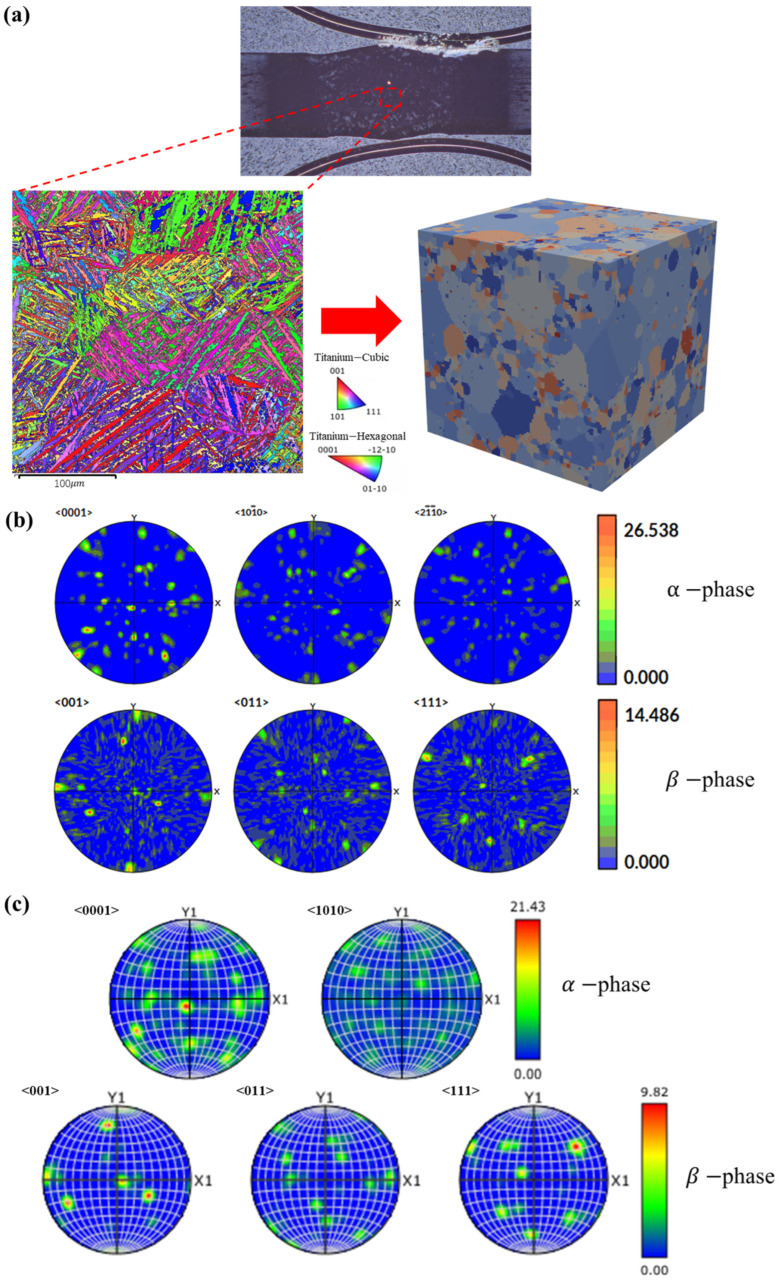
(**a**) RVE morphology; (**b**) RVE’s PF data; (**c**) EBS’s PF data.

The RVE constructed in this study incorporates the authentic microstructure of the TA15 titanium alloy. Based on EBSD scan data from the weld region, the Dream3D software (v6) [24] was employed to perform a statistical analysis of the 2D grain Euler angle orientation information, thereby constructing a 3D representative volume based on its synthetic orientation characteristics. As illustrated in Figure 5a, data samples of metallographic orientation characteristics were acquired via EBSD scanning of the heat-affected zone (HAZ) of the TA15 weld. The scanned area measured 300 μm × 300 μm, capturing a total of 1949 grains. Using this characteristic sample and a synthetic statistical method, the orientation distribution function (ODF), grain shapes, and aspect ratios were calculated. Consequently, a statistics file was generated, which was utilized to create the 3D RVE.

To ensure that the finite element tensile experiments accurately reflect the true mechanical properties of the material, it is essential to determine an appropriate RVE size [25]. Accordingly, a cubic RVE with dimensions of 200 μm × 200 μm × 200 μm was constructed in this study for the tensile simulations. Figure 5b presents the pole figure data of the generated RVE, which exhibits a close approximation to the experimental EBSD pole figure shown in Figure 5c. This comparison indicates that the generated RVE possesses texture information similar to that of the actual material. Consequently, in the subsequent crystal plasticity finite element simulations used to predict the stress–strain curves, the model is capable of effectively capturing the activation of the slip systems during the tensile and shear processes.

The crystal plasticity simulation requires determination of parameters such as the material elastic constants, hardening exponents, CRSS, initial slip resistance, saturation shear stress, and initial self-hardening rate. The elastic constants of the α-phase and β-phase of the TA15 alloy derived from the research of Jin-Yeon Kim et al. [26] using line-focus acoustic microscopy are presented in Table 2. Based on the experimental results of Yanxi Li et al. [27], the plastic constitutive parameters of the TA15 alloy are listed in Table 3 and Table 4; where ${\dot{\gamma }}_{0}$ is the reference shear rate, m is the strain rate sensitivity index, n is the strain hardening exponent, τ is the CRSS, g is the saturated slip strength.

Under these material parameters, the tensile deformation is simulated and the stress–strain curves are outputted. The macroscopic boundary conditions are implemented by controlling the mixed components of the deformation gradient rate tensor $\dot{F}$ and the first Piola–Kirchhoff stress tensor P.

**Figure 6:**
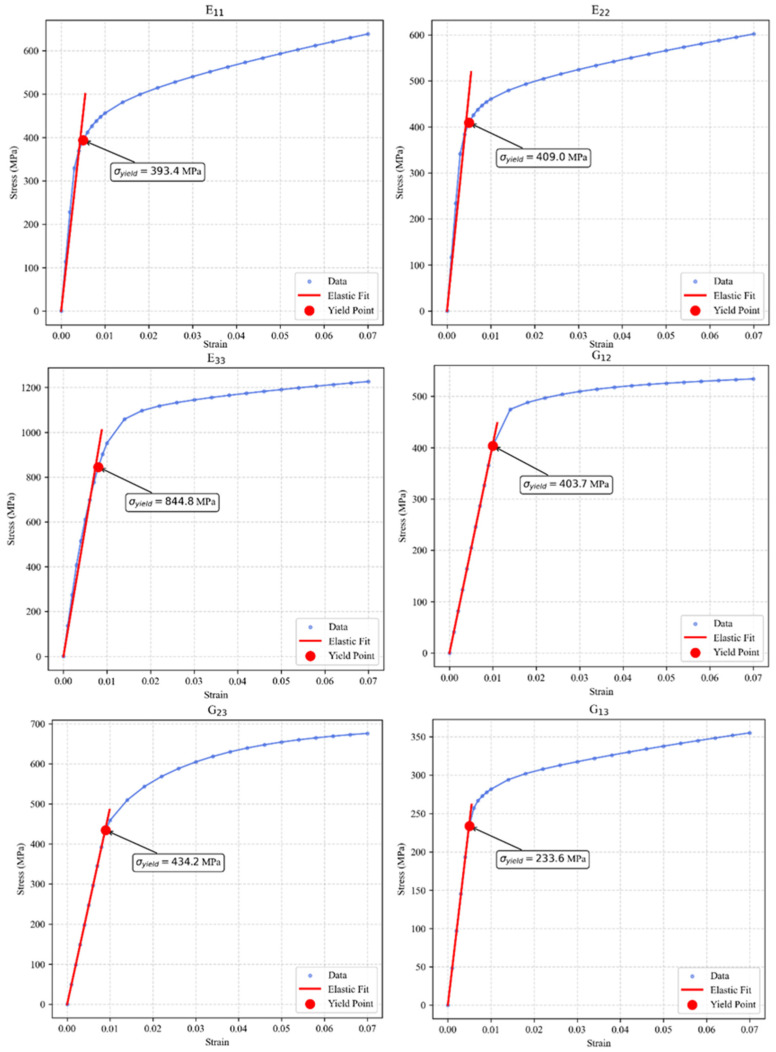
Stress–strain curve of the RVE.

Taking the uniaxial tensile simulation along the positive *X*-axis as a representative case, the tensile strain rate was set to ${\dot{F}}_{11}=1\times 10^{-3}s^{-1}$. To enforce a uniaxial tensile stress state, the stress components along the Y- and Z-axes, perpendicular to the *X*-axis, were prescribed as zero. The simulation was terminated when the macroscopic total tensile strain reached 7%. Similar tensile simulations were subsequently conducted along the Y-axes and Z-axes. Regarding the shear simulations, taking the shear along the XY plane as an example, the shear strain rate component was set to ${\dot{F}}_{12}=1\times 10^{-3}s^{-1}$, while all other components were constrained to zero. This ensured that the RVE underwent isochoric pure shear deformation. Analogous shear simulations were performed for the YZ and XZ planes. Figure 6 presents the stress–strain curves for these six loading directions; the curves were plotted based on data points exported from DAMASK, with the red lines indicating the linear regression fits for the elastic deformation regimes.

By substituting the yield stresses from the various directions into Equations (13)–(18), the values of the constant terms were obtained, as listed in Table 5.

The macroscopic elastic constants and Poisson’s ratios derived from the stress–strain curves are presented in Table 6.

As indicated by the data in Table 6, the value of $E_{z}$ is significantly higher than those of $E_{x}$ and $E_{y}$, suggesting that grain growth within the weld seam possesses a strong preferred orientation. Specifically, the c-axes of the majority of α-phase grains tend to align parallel to the *Z*-axes or exhibit a small inclination angle relative to it. Regarding Poisson’s ratio, the material undergoes pronounced in-plane contraction when subjected to tensile loading along the X- axes or Y-axes. This phenomenon demonstrates that the activation of crystal slip systems is strictly direction dependent.

Substituting the aforementioned parameters into Equation (6) and performing the inverse matrix transformation via Equation (7), the stiffness matrix *D* for the TA15 material at room temperature is obtained as follows:

(29) $$D=\left[\begin{array}{cccccc} 158.1 & 101.5 & 58.5 & 0 & 0 & 0\\ & 160.8 & 59.5 & 0 & 0 & 0\\ & & 182.2 & 0 & 0 & 0\\ & \mathrm{SYM} & & 40.5 & 0 & 0\\ & & & & 49.2 & 0\\ & & & & & 47.2 \end{array}\right](\mathrm{GPa})$$

### 3.3. Thermo-Elasto-Plastic Macroscopic Finite Element

Solid modeling and meshing were performed on two 75 mm×50 mm×2 mm TA15 plates. The 3D finite element model used in this study was constructed using the preprocessing software HyperMesh (V14.0). To ensure computational accuracy and enhance solution efficiency, the model was entirely meshed using three-dimensional, eight-node hexahedral elements.

**Figure 7:**
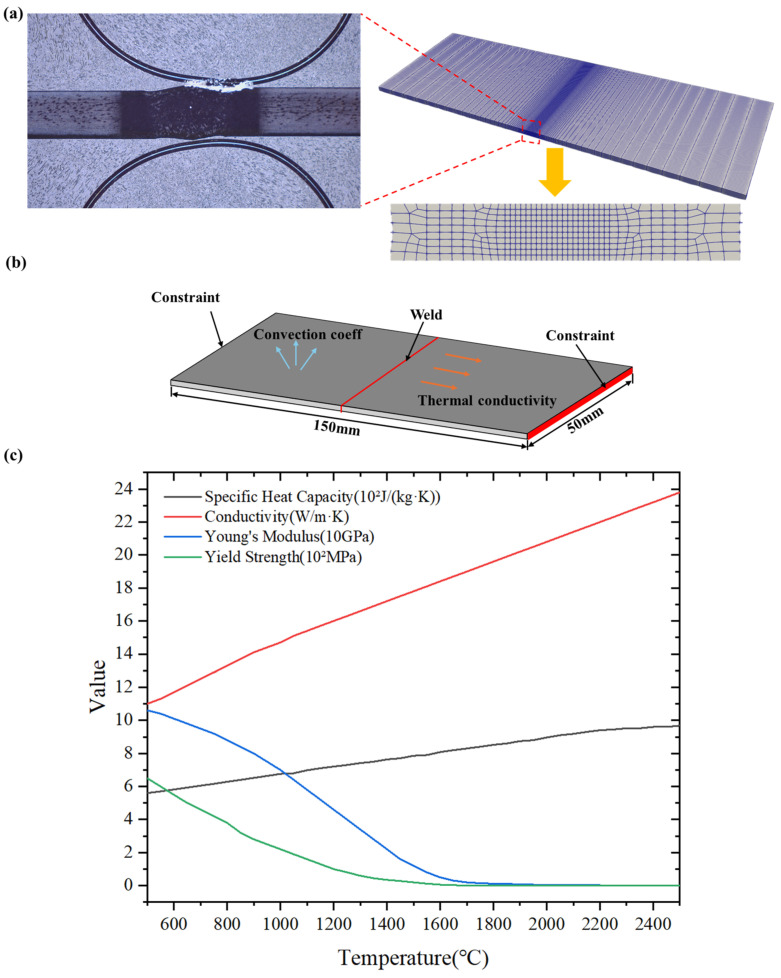
(**a**) Grid division strategy diagram; (**b**) Simulation parameter settings and constraint positions; (**c**) Thermo-elasto-plasticity parameters varying with temperature.

Given the high energy density and steep temperature gradients characteristic of the central weld zone in electron beam welding, a local mesh refinement strategy was implemented for the weld seam and the HAZ to accurately capture the dynamic thermal response, as illustrated in Figure 7a. Conversely, a relatively coarser mesh was adopted for regions subjected to minimal thermal influence. To balance computational efficiency with the capability to capture severe temperature gradients, the determination of mesh size was based on the characteristic length of the heat source. As detailed in Table 7, considering that the minimum semi-axis length of the double-ellipsoid heat source model is 1 mm, the mesh dimensions within the weld core region were set to 0.2 mm×0.2 mm×0.5 mm. This strategy ensures that at least five elements are spanned within the radius of concentrated heat source energy, which is sufficient to accurately describe the energy distribution of the double-ellipsoid model and guarantee the convergence of the numerical solution.

In the experimental welding, the welding input power was 950 W, and the welding torch moving speed was 30 mm/s. The post-weld cross-section is shown in Figure 7a; measurements indicated a weld seam width of approximately 2 mm and a heat-affected zone width of approximately 7 mm.

In the experimental welding process, the welding input power was set to 950 W, and the welding speed was maintained at 30 mm/s. The cross-section of the post-weld joint is illustrated in Figure 7a; measurements indicate a weld width of approximately 2 mm and a HAZ width of approximately 7 mm. Regarding the thermo-elasto-plastic finite element simulation, in addition to defining the experimental power and travel speed, it is essential to determine the specific heat source parameters. The characteristic parameters for the double-ellipsoid heat source model are detailed in Table 7. Furthermore, the simulation accounts for temperature-dependent material properties, including specific heat capacity, Young’s modulus, and yield strength. The boundary constraints applied to the plate are depicted in Figure 7b, while the variations of material parameters with temperature are presented in Figure 7c.

According to the thermophysical property curves shown in Figure 7c, the temperature-dependent Young’s modulus *E*(*T*) and yield strength $\sigma_{y}\left(T\right)$ are substituted into Equations (24)–(28) to obtain the evolution values of the anisotropic constitutive parameters with respect to temperature.

### 3.4. Surface Residual Stress Experiment

**Figure 8:**
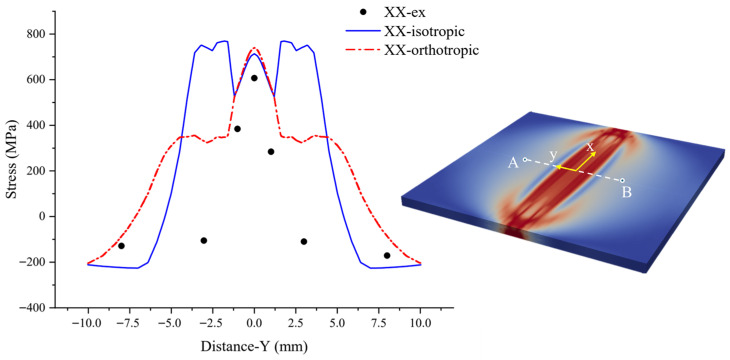
Comparison of surface residual stress distribution.

To characterize the surface residual stress distribution within the weld seam and HAZ of the welded test plate, experimental measurements were conducted using a Proto iXRD X-ray residual stress diffractometer. The sampling strategy and the layout of measurement points are illustrated in Figure 8. The coordinate system was defined such that the *X*-axis is parallel to the welding direction, while the *Y*-axis is perpendicular to it. The measurement path extends along line segment AB, which spans across the center of the weld. With the weld center established as the coordinate origin, residual stress values in the x directions were determined at the following specific locations: y = 0 mm, ±1 mm, ±3 mm, and ±8 mm.

## 4. Results and Discussion

### 4.1. Analysis of Residual Stress on Weld Surface

Figure 8 presents a comparative validation between the finite element simulation results and the experimental measurements of welding residual stress. It is important to note that the data depicts the surface residual stress distributions along the transverse line segment AB (perpendicular to the welding direction on the surface). Specifically, the discrete data points labeled ‘XX-ex’ represent the experimental measurements; the curves ‘XX-isotropic’ denote the numerical simulation results based on the isotropic constitutive model; and the curves ‘XX-orthotropic’ correspond to the numerical simulation results derived from the elastic-plastic orthotropic constitutive model. Given that the RVE in Section 3.2 was constructed based on EBSD data from the weld center region, to ensure consistency between the simulation conditions and the experimental conditions, the interval of −10 mm ≤ Y ≤ 10 mm was selected for analysis.

Regarding the residual stress along the positive *X*-axis, the experimental data and results from both models indicate a tensile stress state in the weld center region (−5 mm < Y < 5 mm), which gradually transitions to compressive stress in the regions where Y ≥ 5 mm and Y ≤ −5 mm. The peak tensile stress $\sigma_{xx}$ predicted by the isotropic model is comparable to that of the orthotropic model, with both reaching approximately 800 MPa. However, the isotropic model exhibits a distinct ‘M’-shaped residual stress distribution within the weld center. This phenomenon arises because the isotropic model treats the material as a homogeneous continuum, neglecting the induction of plastic slip by grain orientation, which leads to an overestimation of stress concentration. Upon incorporating elastic and plastic orthotropic characteristics, the peak stress beyond the fusion line decreases significantly to approximately 350 MPa, and the stress gradients near the fusion line become more gradual, demonstrating higher consistency with the distribution trends observed in the experimental measurement points.

In summary, compared to traditional isotropic models, the orthotropic model based on cross-scale CPFEM coupling exhibits higher fidelity in predicting welding residual stresses. This model effectively rectifies the discrepancies in the residual stress field distribution along the XX directions within the weld center. Furthermore, the average relative error between the numerical simulation results and the experimental data is controlled within a reasonable range.

### 4.2. Validation of Molten Pool Morphology and Temperature Field

**Figure 9:**
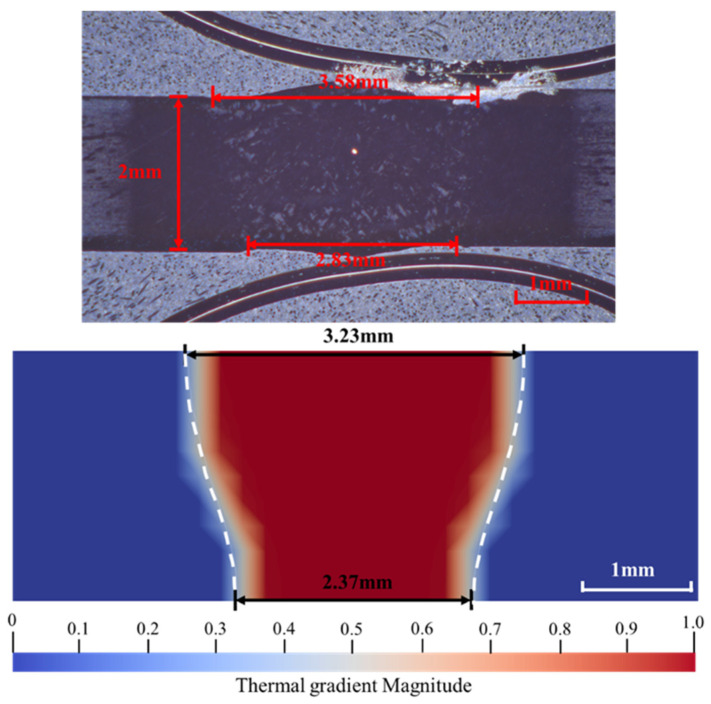
Comparison of molten pool morphology between experiment and simulation.

The computational accuracy of the temperature field significantly influences the precision of the finite element simulation of welding residual stress. Given that the subsequent orthotropic and isotropic models employ the same double-ellipsoidal heat source model and thermal boundary conditions, this section focuses on validating the accuracy of the molten pool morphology calculated under these heat source parameters. Figure 9 presents the comparison results between the experimental metallographic cross-section of the TA15 electron beam welded joint and the molten pool morphology obtained from the finite element simulation.

The experimental macroscopic metallographic morphology reveals that the weld fusion zone exhibits a typical ‘nail-shaped’ profile, confirming that the electron beam achieved full penetration of the plate. Quantitative measurements indicate that the top surface width of the experimental weld is 3.58 mm, while the bottom width is 2.83 mm.

By extracting the temperature gradient field contour map from the simulation results and defining the melting point boundary, indicated by the white dashed line, the experimentally observed geometric characteristics of the molten pool—wider at the top and narrower at the bottom—were successfully reproduced. Quantitative analysis indicates that the simulated top surface width is 3.23 mm, and the bottom width is 2.37 mm. Despite limitations imposed by the idealized assumptions of the heat source model and simplifications of boundary heat dissipation conditions, which resulted in relative errors of approximately 9.8% for the top width and 16.3% for the bottom width, the simulated depth-to-width ratio and the overall nail-shaped weld morphology maintain a high degree of consistency with the experimental results.

The aforementioned results demonstrate that the double-ellipsoid heat source model and its associated shape parameters adopted in this study accurately characterize the heat input features and energy distribution of electron beam welding. Consequently, the calculated transient temperature field exhibits high fidelity, thereby providing reliable thermal boundary conditions for the subsequent macroscopic residual stress calculations.

### 4.3. Analysis of Stress Difference in Weld Cross Section

**Figure 10:**
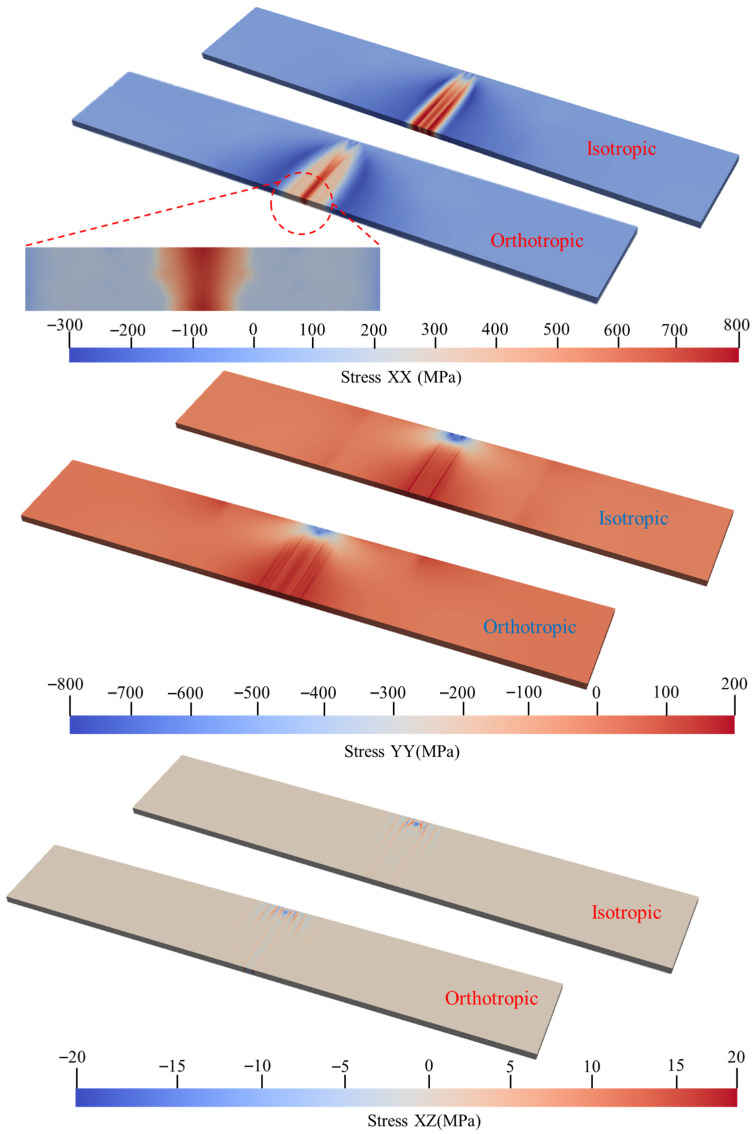
Macroscopic stress comparison.

To investigate the influence of microscopic crystallographic texture on macroscopic mechanical behavior, a comparative evaluation of the overall stress distribution in the welded plate was first performed. Figure 10 illustrates the macroscopic stress fields of the TA15 plate along the *X*-axis, *Y*-axis, and XZ direction under both isotropic and orthotropic constitutive models. The stress field contour plots reveal that in the non-heat-affected zone far from the weld seam, the stress distribution characteristics and numerical magnitudes of the orthotropic model are consistent with those of the isotropic model, indicating that the anisotropic mechanical characteristics of the material are not significant in the non-heat-affected zone.

In contrast, significant discrepancies are observed in the stress field morphologies of the two models within the weld fusion zone and the heat-affected zone. As indicated by the circular region marked in Figure 10, this section focuses the specific analysis of field variables on the transverse region defined by −4.5 mm < Y < 4.5 mm, which is symmetric about the weld center. This characteristic region completely encompasses the fusion zone, characterized by preferred grain orientation growth, as well as a portion of the heat-affected zone.

To elucidate the influence of anisotropic mechanical characteristics induced by microscopic crystallographic texture on the macroscopic mechanical behavior of the weld, this study conducted a comparative analysis between the isotropic model and the CPFEM-based orthotropic model. The analysis focused on the distribution characteristics of the stress fields, elastic strain fields, and plastic strain fields along the longitudinal (XX), transverse (YY), and shear (XZ) directions.

**Figure 11:**
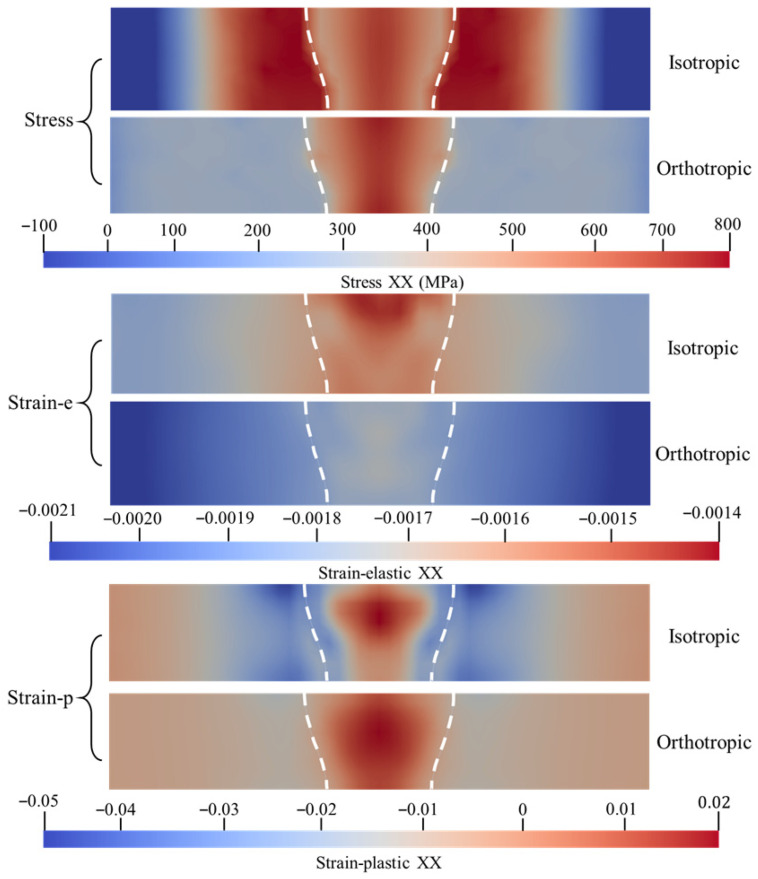
Comparison of stress and strain maps in orthogonal anisotropic and isotropic weld regions along XX.

As illustrated in Figure 11, where the white dashed lines denote the weld fusion lines, the isotropic model exhibits a significant tensile stress concentration zone accompanied by steep stress gradients within the fusion zone along the direction parallel to the welding path (XX direction). The stress concentration is distributed across the weld center and the regions exterior to the fusion lines, presenting a ‘high-low-high-low-high’ ‘M’-shaped distribution pattern. This observation is consistent with the surface residual stress distribution characteristics of the isotropic model in the weld center region discussed in Section 4.1. Although the stress field of the orthotropic model maintains consistency with the isotropic model in terms of overall morphology, the distribution range of the high-stress zone is relatively narrower, and the stress gradients exterior to the fusion lines are more gradual. In terms of overall magnitude, the peak longitudinal residual stress predicted by the orthotropic model is slightly lower than that of the isotropic model.

From the perspective of elastic and plastic strain fields, the two models exhibit even more pronounced distinctions. Regarding elastic strain distribution, the orthotropic model presents a lower level of elastic strain at the weld center, whereas the isotropic model generates substantial elastic strain in the weld center region adjacent to the plate surface. Concerning plastic flow behavior, results from the orthotropic model indicate that plastic flow is concentrated within the weld center region, with negligible plastic deformation occurring exterior to the fusion lines. In contrast, the isotropic model demonstrates a broader zone of concentrated plastic strain with higher magnitudes; in addition to the plastic tensile strain at the weld center, extensive plastic compressive deformation is also observed in the vicinity of the fusion lines.

**Figure 12:**
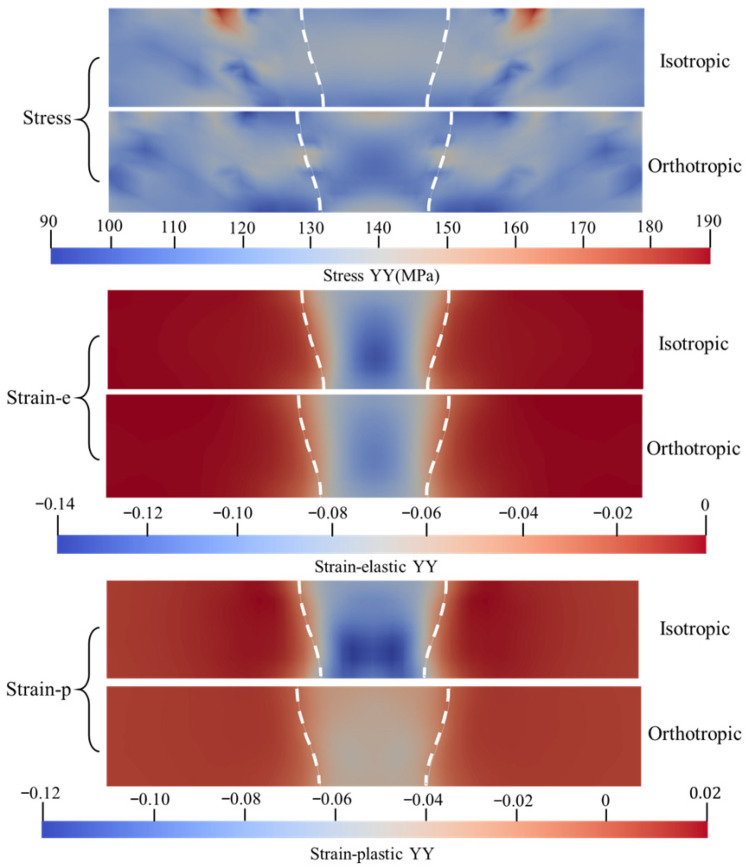
Comparison of stress and strain maps in orthogonal anisotropic and isotropic weld regions along YY.

As illustrated in Figure 12, in the direction perpendicular to the welding path (YY direction), the isotropic model exhibits a relatively continuous and uniform tensile stress band within the center of the weld fusion zone. The stress values transition smoothly between 94 MPa and 150 MPa, with a significant peak tensile stress observed at the weld surface. In contrast, the orthotropic model displays pronounced fluctuation characteristics, characterized by an alternating distribution pattern of high and low stresses within the fusion line and the fusion zone. Overall, the average stress magnitude of the isotropic model is higher than that of the orthotropic model.

In terms of elastic and plastic strain fields, the isotropic and orthotropic models exhibit similar distribution patterns, with both elastic deformation and plastic flow occurring within the fusion zone. However, the isotropic model predicts a concentrated zone of large-magnitude compressive plastic strain at the weld center, indicating that the material underwent severe transverse compressive yielding during the heating phase of the welding thermal cycle. Conversely, the plastic strain values predicted by the orthotropic model in this same region are close to zero, suggesting that the weld underwent negligible plastic deformation in the transverse direction according to this prediction.

Based on the yield data presented in Figure 6, the XZ shear plane exhibits the lowest yield threshold, making it the preferred orientation for the initiation of plastic slip. Given the high sensitivity of this direction to plastic flow, the XZ direction was selected to analyze the significant discrepancies in the mechanical response predictions between the orthotropic and isotropic models.

**Figure 13:**
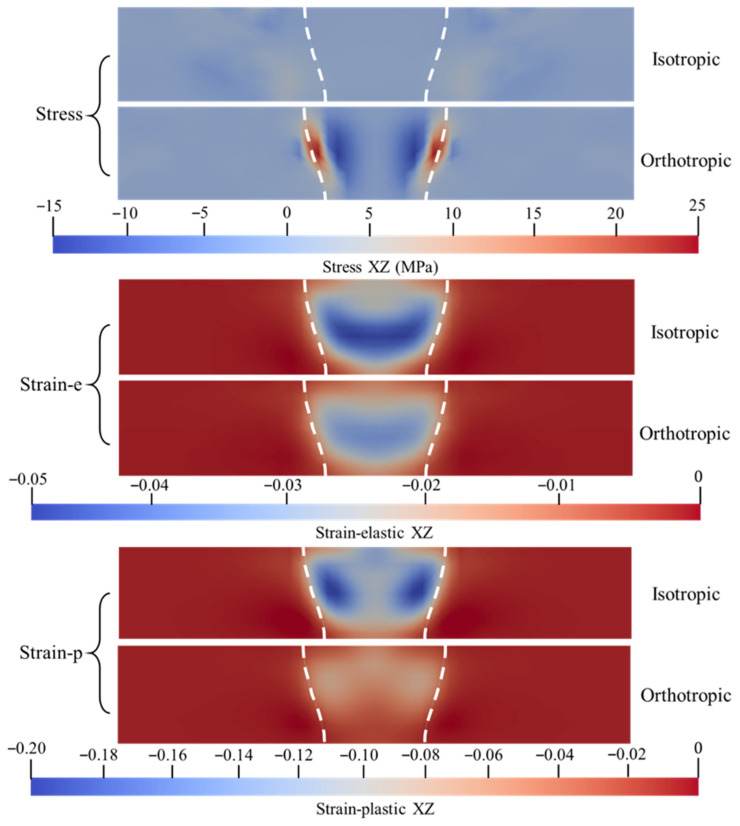
Comparison of stress and strain maps in orthogonal anisotropic and isotropic weld regions along XZ.

Figure 13 presents the map of the shear component in the XZ direction within the transverse cross-section of the weld. Regarding the stress field distribution, the isotropic model exhibits a uniform low-stress state throughout the entire weld seam and heat-affected zone. The values approach zero, indicating that the model fails to capture any significant shear stress components. In contrast, the orthotropic model predicts distinct shear stress concentration bands at the boundaries on both sides of the fusion line. These bands display a symmetrical ‘wing-like’ distribution pattern, with local peak stresses significantly elevated to approximately 25 MPa.

The results of the elastic strain field reveal that the isotropic model forms a large-scale zone of concentrated compressive strain at the weld center. In contrast, the corresponding elastic strain region in the orthotropic model is significantly contracted, exhibiting a more flattened morphology with gentler internal numerical gradients. Regarding the plastic strain field, the isotropic model predicts two symmetrical, high-magnitude plastic shear slip zones within the weld, indicating the occurrence of severe localized plastic deformation in this region. Conversely, in the orthotropic model, the plastic shear strain values throughout the entire weld zone remain at a near-zero level with a relatively uniform distribution. This result highlights a fundamental distinction between the two models in predicting plastic flow behavior along the shear direction.

Based on the comprehensive comparative analysis of the field variables described above, it is evident that the orthotropic finite element model, grounded in cross-scale CPFEM coupling, demonstrates significant superiority in predicting the complex mechanical behavior of welding. Compared to traditional isotropic models, this model successfully captures the unique mechanical response characteristics of the weld zone by incorporating the homogenized stiffness matrix D and Hill yield criterion parameters, which characterize microscopic crystallographic texture features. Consequently, this approach significantly enhances the prediction accuracy and fidelity of the residual stress fields in electron beam welding. This model not only facilitates the more accurate identification of high-risk zones for potential crack initiation within welded joints but also provides a more rigorous theoretical basis and data support for optimizing welding process parameters to control deformation, evaluating the in-service reliability of welded structures, and formulating targeted residual stress mitigation strategies.

## 5. Conclusions

This study addresses the challenge of accurately predicting welding residual stresses in titanium alloys characterized by strong crystallographic textures. By bridging the gap between micro-scale crystal plasticity and macro-scale thermo-elasto-plastic finite element analysis, a higher fidelity stress simulation algorithm was established. This algorithm integrates the CPFEM with thermo-elasto-plastic theory to calculate the homogenized orthotropic stiffness matrix and Hill yield criterion parameters. It successfully characterizes the influence of grain texture features within the weld zone on mechanical properties and extends this characterization to macroscopic numerical calculations, thereby overcoming the accuracy bottlenecks associated with traditional isotropic models that neglect microstructure-induced anisotropy.

The high fidelity of the proposed orthotropic model in predicting residual stress was rigorously validated. Numerical simulation results demonstrate that the model maintains the prediction error of the molten pool dimensions within 16.3%. More importantly, the model effectively corrects the overestimation of longitudinal tensile stress inherent in traditional isotropic models at the weld fusion line, adjusting the peak value from approximately 800 MPa to 350 MPa, which is consistent with experimental data. Furthermore, it successfully captures a shear stress component of approximately 25 MPa on the XZ shear plane, which is typically missed by isotropic formulations.

In conclusion, these findings suggest that characterizing micro-texture features by calculating the stiffness matrix and Hill yield criterion constants is of crucial significance for mitigating systematic bias in stress prediction within electron beam welding numerical simulations. This work realizes a coupled algorithm that derives material constitutive equations through crystal plasticity solutions, providing a robust theoretical basis for optimizing welding processes and assessing the reliability of critical aerospace components.

## Figures and Tables

**Table 1:** Chemical composition of TA15.

Element	O	Zr	Mo	V	Fe	Al	Ti
wt%	0.015	2.15	1.24	1.73	0.045	6.45	Balance

**Table 2:** Elastic constants of single crystal α-phase and β-phase.

	$C_{11}$	$C_{33}$	$C_{12}$	$C_{13}$	$C_{44}$
α-phase (GPa)	141	163	76.9	57.9	48.78
β-phase (GPa)	135		113		54.9

**Table 3:** The parameters of single crystal α-phase deformation CP constitutive model for titanium alloys.

Parameters	${\dot{\gamma }}_{0}^{\alpha }$	m	n	τ	g
Basal	0.0023	0.02	0.15	272 MPa	450 MPa
Prismatic-<a>	0.0023	002	0.15	148 MPa	550 MPa
Pyramidal-<a>	0.0023	0.02	0.05	175 MPa	520 MPa
Pyramidal-<c + a>	0.0023	0.02	0.05	820 MPa	990 MPa

**Table 4:** The parameters of single crystal β-phase deformation CP constitutive model for titanium alloys.

Parameters	${\dot{\gamma }}_{0}^{\beta }$	m	τ	g
{101}	0.0023	0.02	330 MPa	550 MPa

**Table 5:** Parameters of Hill anisotropic yield criterion.

$F\;({MPa}^{-2}$)	$G\;({MPa}^{-2}$)	$H\;({MPa}^{-2}$)	$L\;({MPa}^{-2}$)	$M\;({MPa}^{-2}$)	$N\;({MPa}^{-2}$)
${4.588\times 10}^{-7}$	${9.423\times 10}^{-7}$	${5.519\times 10}^{-6}$	${2.652\times 10}^{-6}$	${9.163\times 10}^{-6}$	${3.068\times 10}^{-6}$

**Table 6:** Elastic constant and Poisson’s ratio.

E		v	
$E_{x}$	90.9 GPa	$v_{12}$	0.529
$E_{y}$	94.3 GPa	$v_{13}$	0.237
$E_{z}$	127.7 GPa	$v_{21}$	0.549
$G_{xy}$	40.7 GPa	$v_{23}$	0.207
$G_{xz}$	47.6 GPa	$v_{31}$	0.333
$G_{yz}$	49.3 GPa	$v_{32}$	0.281

**Table 7:** Characteristic parameters of the double ellipsoidal heat source model.

Parameters	Q	a	b	$c_{1}$	$c_{2}$	$f_{f}$	$f_{r}$
Value	950 W	1.5 mm	2 mm	1 mm	2 mm	1	1

## Data Availability

The original contributions presented in this study are included in the article. Further inquiries can be directed to the corresponding author.
